# Emergence of the M phenotype of erythromycin-resistant pneumococci in South Africa.

**DOI:** 10.3201/eid0402.980216

**Published:** 1998

**Authors:** C A Widdowson, K P Klugman

**Affiliations:** South African Institute for Medical Research, Johannesburg, South Africa. 174carol@chiron.wits.ac.za

## Abstract

Erythromycin-resistant pneumococci have been isolated in South Africa since 1978; however, from 1987 to 1996, resistance to macrolides was only detected in 270 (2.7%) of 9,868 blood or cerebrospinal fluid (CSF) pneumococcal isolates, most of which were obtained from the public sector. In South Africa, macrolide use in the public sector is estimated at 56% of that in the private sector. Most erythromycin-resistant strains (89%) exhibited resistance to erythromycin and clindamycin (macrolide-lincosamide-streptogramin B phenotype). In the United States, most erythromycin-resistant pneumococci exhibit the newly described M phenotype (resistance to erythromycin alone), associated with the mefE gene. The M phenotype in South Africa increased significantly in the last 10 years, from 1 of 5,115 to 28 of 4,735 of blood and CSF isolates received from 1987 to 1991 compared with 1992 to 1996 (p = 5 x 10(-7)). These data suggest that, although macrolide resistance in pneumococci remains low in the public sector, the mefE gene is rapidly emerging in South Africa.


### Emergence of the M Phenotype of Erythromycin-Resistant Pneumococci in South Africa

**Carol A. Widdowson and Keith P. Klugman**
South African Institute for Medical Research, Johannesburg, South Africa

Erythromycin-resistant pneumococci have been isolated in South Africa since 1978; however, from 1987 to 1996, resistance to macrolides was only detected in 270 (2.7%) of 9,868 blood or cerebrospinal fluid (CSF) pneumococcal isolates, most of which were obtained from the public sector. In South Africa, macrolide use in the public sector is estimated at 56% of that in the private sector. Most erythromycin-resistant strains (89%) exhibited resistance to erythromycin and clindamycin (macrolide-lincosamide-streptogramin B phenotype). In the United States, most erythromycin-resistant pneumococci exhibit the newly described M phenotype (resistance to erythromycin alone), associated with the *mefE* gene. The M phenotype in South Africa increased significantly in the last 10 years, from 1 of 5,115 to 28 of 4,735 of blood and CSF isolates received from 1987 to 1991 compared with 1992 to 1996 ($p = 5x10^{-7}$). These data suggest that, although macrolide resistance in pneumococci remains low in the public sector, the *mefE* gene is rapidly emerging in South Africa.

Resistance to erythromycin in pneumococci has been observed since 1967 (1) and was first reported in South African multiresistant pneumococcal strains in 1978 (2). Until recently, the only mechanism described for resistance to erythromycin in the pneumococcus was the $N^6$-methylation of a specific adenine residue (A2058) in 23S rRNA, which resulted in reduced affinity between the antibiotic and the ribosome (3,4). This resistance is associated with the gene *ermAM* (5), first described in *Streptococcus sanguis* (6). Since then, other mechanisms of erythromycin resistance in the pneumococcus have been reported. In fact, most resistance in the United States appears to be due to efflux of the antibiotic from the cell, associated with the gene *mefE* (7,8). While *ermAM* confers coresistance to most macrolides, lincosamides, and streptogramin B antibiotics (resulting in the so-called MLS phenotype) (3,9), *mefE* confers resistance only to the 14- and 15-membered macrolides (resulting in the M phenotype) (7,8). We report the emergence of M-phenotype erythromycin resistance in South African blood and cerebrospinal fluid (CSF) pneumococcal isolates from 1987 to 1996.

The South African Institute for Medical Research (SAIMR), Johannesburg, South Africa, regularly receives all pneumococcal isolates from participating laboratories in eight of the nine provinces of South Africa. We examined all erythromycin-resistant blood and CSF isolates received by SAIMR from 1987 to 1996.

Erythromycin-, clindamycin-, and penicillin-resistance phenotypes were determined by using disk diffusion assays (erythromycin, 15µg/disk, clindamycin, 2 µg/disk, oxacillin, 1 µg/disk) on 5% horse blood agar plates (Mueller-Hinton base) after overnight growth at 37°C under aerobic conditions. Strains showing resistance (zone diameters ≤ 20mm for erythromycin, ≤ 18mm for clindamycin, and <20mm for oxacillin) on the disk diffusion plates were tested by the agar dilution method to obtain MICs according to the National Committee for Clinical Laboratory Standards guidelines (10). We evaluated (by the chi-square test) increases in the prevalence of erythromycin resistance and the incidence of resistance to erythromycin and susceptibility to clindamycin, which represents the M phenotype.

The M phenotype increased significantly from 1987 to 1991 compared with 1992 to 1996 in

Address for correspondence: Carol A. Widdowson, Department of Clinical Microbiology and Infectious Diseases, SAIMR, P.O. Box 1038, Johannesburg 2000, South Africa; fax: 27-11-489-9332; e-mail: 174carol@chiron.wits.ac.za.

blood and CSF isolates—from 1 of 5,115 isolates to 28 of 4,753 isolates ($p = 5x10^{-7}$, odds ratio [OR] 30.13, 95% confidence interval [CI] 4.1-221) (Table 1; Figure).

Data on oral macrolides in the public sector (from the Division of Medical Schemes Supplies and Pharmaceutical Services of the Department

Table 1. Prevalence of South African erythromycin-resistant pneumococcal isolates, 1987–1996[a]

| Years | Total isolates[b] | No. of E-R strains (%)[c] | No. of M strains (% of E-R strains) |
|---|---|---|---|
| 1987-1991 | 5,115 | 128 (2.5) | 1 (0.8) |
| 1992-1996 | 4,753 | 142 (3.0) | 28 (19.7) |
| Total | 9,868 | 270 (2.7) | 29 (10.7) |

[a]Of 9,868 blood and cerebrospinal fluid (CSF) isolates received by the SAIMR from 1987 to 1996, 270 were fully resistant to erythromycin. While the number of erythromycin-resistant blood and CSF isolates received increased from 1987 to 1991 compared with 1992 to 1996 (2.5% to 3.0%), the increase was not significant. There was no significant relationship between erythromycin resistance and the M phenotype within any given province throughout the 10 years.
[b]Blood and cerebrospinal fluid isolates.
[c]E-R= erythromycin-resistant.

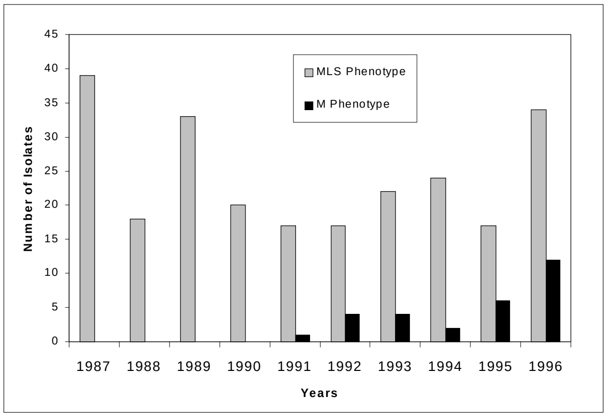

Figure. Number of erythromycin-resistant blood and cerebrospinal fluid isolates of pneumococci.

of Health) show that 16.4 million defined daily doses (ddd) of macrolides were purchased for the estimated 30.3 million persons who obtain health care from the public sector (0.54 ddd per capita). Private sector use for the year ending December 1997 (Intercontinental Medical Statistics South Africa, Pty, Ltd., unpub. data) show that 7.3 million ddd of macrolide were purchased in an estimated population of 7.57 million (0.96 ddd per capita).

All 78 MLS isolates hybridized with the *ermAM* probe or produced a 616-bp-amplification product during polymerase chain reaction (PCR) amplification using the *ermAM*-specific primers.[1] The 12 M isolates tested contained the *mefE* gene as shown by a 348bp-amplification product when amplified using primers specific for *mefE*. There were no erythromycin-resistant isolates that contained neither the *ermAM* nor the *mefE* gene.

Erythromycin-resistant strains were serotyped by using the quellung reaction and antisera from the Staten Seruminstitut, Copenhagen, Denmark. Over the 10 years, the five most common serotypes and groups among the erythromycin-resistant isolates in decreasing order of frequency were serotype 14, serogroups 6, 19, 23, and serotype 1 (Table 2). Serotype 1 erythromycin-resistant pneumococci appeared only after 1992; serotype 14 was the most common in MLS isolates; serogroup 23 was the most common serogroup in M isolates (Table 2). Serotypes 14 and serogroups 6, 23, and 19 are the most common serotypes and groups isolated from children with serious infections (13,14). Of the 157 isolates from patients whose age was supplied, 98 (62%) were obtained from children (≤12 years). There was a trend that was not significant toward more macrolide resistance in children than adults (OR 1.17 [95% CI 0.98-1.39]). This trend may have been significant if age data had been supplied with all the isolates received. The proportion of

[1]DNA was extracted from pneumococcal isolates by using a lysis solution consisting of 0.1% sodium deoxycholate as described in (11), except that we used plate rather than broth cultures.

Seventy-eight MLS strains were probed for the *ermAM* gene by using dot blots. The probe (supplied by P. Courvalin, Pasteur Institute, Paris, France) (*Escherichia coli* JM83/pUC19 560bp *Ssp*1 intragenic fragment of *ermB*) was labeled with digoxygenin by using random primed labeling (DIG DNA Labeling and Detection Kit; Boeringer, Mannheim, Germany). Hybridization and detection were performed following manufacturer's instructions (DIG DNA Labeling and Detection Kit; Boeringer, Mannheim, Germany). PCR was also used to detect *ermAM* in 30 strains according to standard conditions, with an annealing temperature of 58°C. We used the following primers: forward primer, 5'-CGAGTGAAAAAGTACTCAACC, reverse primer, 5'-GGCGTGTTTCATTGCTTGATG).

Published primers for the *mefE* gene (5'-AGTATCATTAATCACTAGTGC, and 5'-TTCTTCTGGTACTAAAAGTGG) (12) were used to detect *mefE* through PCR amplification in 13 M strains. Amplification was performed in a Perkin Elmer Cetus DNA Thermal Cycler under standard reaction conditions, with an annealing temperature of 56ºC.

Table 2. Serotype distribution among erythromycin-resistant pneumococci

| Serotype/ group | No. (%) of MLS[a] isolates | No. (%) of M isolates | Total no. (%) of isolates |
|---|---|---|---|
| 14 | 96 (39.8) | 9 (31.0) | 105 (38.9) |
| 6 | 71 (29.5) | 3 (10.3) | 74 (27.4) |
| 19 | 36 (14.9) | 3 (10.3) | 39 (14.4) |
| 23 | 26 (10.8) | 10 (34.5) | 36 (13.3) |
| 1 | 7 (2.9) | 1 (3.5) | 8 (3.0) |
| Other | 5 (2.1) | 3 (10.3) | 8 (3.0) |
| Total | 241 | 29 | 270 |

[a]MLS, macrolides-lincosamides-streptogramin B.

pediatric isolates did not change significantly over the 10-year period (63% during 1987 to 1991 and 62% the 1992 to 1996 period).

Approximately half of all the macrolide-resistant isolates were also either intermediate or fully resistant to penicillin (60 of the 128 isolates from the 1987 to 1991 period, and 72 of the 142 isolates from the 1992 to 1996 period). There was a trend (not significant) toward greater resistance to penicillin in MLS strains. Of the 78 strains with MICs available, 38 (49%) were fully resistant (MIC ≥ 2 µg/ml) to penicillin, while the rest showed intermediate resistance (1 µg ≤ MIC ≤ 0.12 µg). Previous data have indicated that penicillin-intermediate resistance is far more common in South Africa than full resistance to penicillin (15). In 1992, Friedland and Klugman (15) reported that only 3 of 35 penicillin-resistant strains showed high-level resistance.

Coresistance to penicillin limits the use of most macrolides as treatment of penicillin-resistant pneumococcal infections. New semisynthetic macrolides such as the ketolide RU 64004 (16) are being developed, however, that do not show cross-resistance to penicillin or to erythromycin.

Compared with total erythromycin resistance (middle ear fluid, blood, and CSF) in Europe (17-19), the United Kingdom (18), and the United States (19,21,22), the overall prevalence of macrolide resistance is low. In Europe erythromycin resistance varies by country. In Slovakia, almost all pneumococcal isolates are resistant (17), whereas in Portugal only 0.6% of isolates are resistant and the proportion appears to be declining (23). In the United Kingdom, erythromycin resistance increased from 3.3% to 8.6% between 1989 and 1992 in England and Wales (20). In the United States, 10% of pneumococcal isolates appear to be erythromycin resistant (21). Most isolates received by SAIMR are from the public sector, where macrolides are not normally prescribed for pneumococcal infections. Only 4 of the 128 erythromycin-resistant isolates from 1987 to 1991 and 14 of the 142 erythromycin-resistant isolates from 1992 to 1996 were from the private sector. Resistance data from the private sector may show much higher levels of macrolide resistance, a contention supported by previous South African resistance data (1986), where the carriage rates of multiresistant pneumococci were 17.7% in children from more affluent communities and 0% in children from less affluent areas (24). MIC data were available for 15 of the 20 multiresistant isolates, and all 15 were fully resistant to both erythromycin and clindamycin (24).

Before the M phenotype was observed, erythromycin resistance was assumed to indicate cross-resistance to lincosamides and streptogramin B antibiotics in the pneumococcus. The increase in the incidence of M phenotype may warrant investigation into the use of these antibiotics for the treatment of pneumococcal infections. Sutcliffe et al. (7) suggested that clindamycin be considered for the treatment of bacteremia and middle ear and sinus infections caused by *Streptococcus pneumoniae*. Treatment with clindamycin is feasible only if infection with gram-negative pathogens has been excluded and if the *S. pneumoniae* phenotype is known because the strain may show MLS resistance and studies indicate that many penicillin-resistant strains are also clindamycin-resistant (16,25). Visalli and colleagues (25) found that clindamycin concentrations of only 0.06 µg/ml were required to inhibit 90% of penicillin-susceptible strains when grown in air, while clindamycin concentrations of >64 µg/ml were required to inhibit 90% of penicillin-intermediate and -resistant strains.

Studies of streptogramin use against pneumococci show some promise. The streptogramin RP 59500, a mixture of type A streptogramin, dalfopristin, and type B streptogramin quinupristin, is active against pneumococci regardless of their susceptibilities to penicillin or erythromycin (26,27). In contrast to erythromycin, RP 59500 is rapidly bactericidal (26). Clinical and bacteriologic failure has, however, already been reported using pristinamycin (28), an oral streptogramin combination from which RP 59500 was derived.

The M phenotype is thus relatively new in South African pneumococci but is emerging as an important factor in erythromycin-resistant pneumococci. Although the low overall rate of resistance makes the use of streptogramins and lincosamides potentially more feasible for the treatment of pneumococcal infections, coresistance to penicillin and the present high rate of MLS resistance necessitate antibiotic susceptibility testing before these antibiotics are administered.

### Acknowledgments

The authors thank Patrice Courvalin for the probe used in this study, Thora Capper for assistance with MICs, Avril Wasas for performing serotyping, and Robyn Hubner for assistance with data analysis.

This work was supported by the Medical Research Council, the South African Institute for Medical Research, and the University of the Witwatersrand.

Carol Widdowson is completing her Ph.D. at the South African Institute for Medical Research, through the University of the Witwatersrand. Her research focuses mainly on resistance to the nonbetalactam antibiotics such as erythromycin, tetracycline, chloramphenicol, and streptomycin, in the pneumococcus.

Keith Klugman is the director of the South African Institute for Medical Research. He also heads the Pneumococcal Research Unit of the Medical Research Council, the South African Institute for Medical Research, and the University of the Witwatersrand. He has an interest in all aspects of pneumococcal research.

### References

1. Dixon JMS. Pneumococcus resistant to erythromycin and lincomycin. Lancet 1967;i:573.
2. Jacobs MR, Koornhof HJ, Robins-Browne RM, Stevenson CM, Vermaak ZA, Freiman I, et al. Emergence of multiply resistant pneumococci. N Engl J Med 1978;299:735-40.
3. Lai C-J, Weisblum B. Altered methylation of ribosomal RNA in an erythromycin-resistant strain of *Staphylococcus aureus*. Proc Natl Acad Sci U S A 1971;68:856.
4. Weisblum B. Erythromycin resistance by ribosome modification. Antimicrob Agents Chemother 1995;39:577-85.
5. Trieu-Cuot P, Poyart-Salmeron C, Carlier C, Courvalin P. Nucleotide sequence of the erythromycin resistance gene of the conjugative transposon Tn*1545*. Nucleic Acids Res 1990;18:3660.
6. Horinouchi S, Byeon WH, Weisblum B. A complex attenuator regulates inducible resistance to macrolides, lincosamides, and streptogramin type B antibiotics in *Streptococcus sanguis*. J Bacteriol 1983;154:1252-62.
7. Sutcliffe J, Tait-Kamradt A, Wondrack L. *Streptococcus pneumoniae* and *Streptococcus pyogenes* resistant to macrolides but sensitive to clindamycin: a common resistance pattern mediated by an efflux system. Antimicrob Agents Chemother 1996;40:1817-24.
8. Tait-Kamradt A, Clancy J, Cronan M, Dib-Hajj F, Wondrack L, Yuan W, et al. *mefE* is necessary for the erythromycin-resistant M phenotype in *Streptococcus pneumoniae*. Antimicrob Agents Chemother 1997;41:2251-5.
9. Fernandez-Muñoz R, Monro RE, Torres-Pinedo R, Vasquez D. Substrate- and antibiotic-binding sites at the peptidyl-transferase centre of *Escherichia coli* ribosomes. Studies on the chloramphenicol, lincomycin and erythromycin sites. Eur J Biochem 1971;23:185-93.
10. National Committee for Clinical Laboratory Standards. Performance standards for antimicrobial disc susceptibility tests. 6th ed. Wayne (PA): The Committee; 1997.
11. Paton JC, Berry AM, Lock RA, Hansman D, Manning PA. Cloning and expression in *Escherichia coli* of the *Streptococcus pneumoniae* gene encoding pneumolysin. Infect Immun 1986;54:50-5.
12. Sutcliffe J, Grebe T, Tait-Kamradt A, Wondrack L. Detection of erythromycin-resistant determinants by PCR. Antimicrob Agents Chemother 1996;40:2562-6.
13. Robbins JB, Austrian R, Lee C-J, Rastogi SC, Schiffman G, Henrichsen J, et al. Considerations for formulating the second generation pneumococcal capsular polysaccharide vaccine with emphasis on the cross-reactive types within groups. J Infect Dis 1983;148:1136-59.
14. Gray BM, Converse GM III, Dillon HC Jr. Epidemiologic studies of *Streptococcus pneumoniae* in infants: acquisition, carriage, and infection during the first 24 months of life. J Infect Dis 1980;142:923-33.
15. Friedland IR, Klugman KP. Failure of chloramphenicol therapy in penicillin-resistant pneumococcal meningitis. Lancet 1992;339:405-8.
16. Ednie LM, Spangler SK, Jacobs MR, Appelbaum PC. Susceptibilities of 228 penicillin- and erythromycin-susceptible and -resistant pneumococci to RU 64004, a new ketolide, compared with susceptibilities to 16 other agents. Antimicrob Agents Chemother 1997;41:1033-6.
17. Reichler MR, Rakovsky J, Sobotová A, Sláciková M, Hlavácová B, Hill B, et al. Multiple antimicrobial resistance of pneumococci in children with otitis media, bacteremia, and meningitis in Slovakia. J Infect Dis 1995;171:1491-6.
18. Gür D, Ünal S, Akalin HE. Resistance patterns in Turkey. Internat J Antimicrob Agents 1995;6:23-6.
19. Goldstein FW, Acar JF, The Alexander Project Collaborative Group. Antimicrobial resistance among lower respiratory tract isolates of *Streptococcus pneumoniae*: results of a 1992-93 Western Europe and USA collaborative study. J Antimicrob Chemother 1996;38:71-84.
20. Aszkenasy OM, George RC, Begg NT. Pneumococcal bacteraemia and meningitis in England and Wales 1982 to 1992. Commun Dis Rep CDR Rev 1995;5:R45-R50.
21. Doern GV, Brueggemann AB, Holley Jr HP, Rauch AM. Antimicrobial resistance of *Streptococcus pneumoniae* recovered from outpatients in the United States during the winter months of 1994 to 1995: results of a 30-year national surveillance study. Antimicrob Agents Chemother 1996;40:1208-13.

22. Butler JC, Hofmann J, Cetron MS, Elliott JA, Facklam RR, Breiman RF, et al. The continued emergence of drug-resistant *Streptococcus pneumoniae* in the United States: an update from the Centers for Disease Control and Prevention's Pneumococcal Surveillance System. J Infect Dis 1996;174:986-93.
23. Vaz Pato MV, Belo de Carvalho C, Tomasz A, the Multicenter Study Group. Antibiotic susceptibility of *Streptococcus pneumoniae* isolates in Portugal. A multicenter study between 1989 and 1993. Microb Drug Resist 1995;1:59-69.
24. Klugman KP, Koornhof HJ, Kuhnle V. Clinical and nasopharyngeal isolates of unusual multiply resistant pneumococci. The American Journal of Diseases of Children 1986;140:1186-90.
25. Visalli MA, Jacobs MR, Appelbaum PC. Susceptibility of penicillin-susceptible and -resistant pneumococci to dirithromycin compared with susceptibilities to erythromycin, azithromycin, clarithromycin, roxithromycin, and clindamycin. Antimicrob Agents Chemother 1997;41:1867-70.
26. Pankuch GA, Lichtenberger C, Jacobs MR, Appelbaum PC. Antipneumococcal activities of RP 59500 (quinupristin-dalfopristin), penicillin G, erythromycin, and sparfloxacin determined by MIC and rapid time-kill methodologies. Antimicrob Agents Chemother 1996;40:1653-6.
27. Barry AL, Fuchs PC. In vitro activities of a streptogramin (RP59500), three macrolides, and an azalide against four respiratory tract pathogens. Antimicrob Agents Chemother 1995;39:238-40.
28. Burucoa C, Pasdeloup T, Chapon C, Fauchère JL, Robert R. Failure of pristinamycin treatment in a case of pneumococcal pneumonia. Eur J Clin Microbiol Infect Dis 1995;14:341-2.
